# The clinical value of Cardiovascular Magnetic Resonance in patients with MR-conditional pacemakers

**DOI:** 10.1186/1532-429X-17-S1-P360

**Published:** 2015-02-03

**Authors:** Claire E Raphael, Francisco Alpendurada, Sanjay K Prasad, Dudley J Pennell, Raad Mohiaddin

**Affiliations:** 1 Department of CMR, Royal Brompton Hospital, London, UK

## Background

Magnetic resonance (MR) conditional pacemakers are increasingly implanted into patients who may need cardiovascular MR (CMR) subsequent to device implantation. While the safety profile appears good, limited data exists regarding the indication and outcome of these examinations for clinical management.

## Methods

CMR and pacing data from consecutive patients with MR conditional pacemakers performed at our centre was retrospectively reviewed. Images were acquired at 1.5T (Siemens Magnetom Avanto). The indication for CMR and any resulting change in management was recorded. The quality of CMR was rated by an observer blinded to clinical details and data on pacemaker and lead parameters collected pre- and post- CMR.

## Results

45 CMR scans on 42 patients performed between 2011 and 2014 were assessed. All scans were completed successfully with no significant change in lead thresholds or pacing parameters. Steady state free precession (SSFP)cines were non-diagnostic in 6/45 (13%) scans, but diagnostic in all patients when using gradient echo sequences (GRE). Late gadolinium enhancement imaging was performed in 39 patients and appeared less susceptible to artefact than cine images.The CMR data resulted in a new diagnosis in 19 (42%) of examinations, clinical management was changed in a further 14 (31%).

## Conclusions

CMR in patients with MR conditional pacemakers provided diagnostic or management-changing information in the majority (73%) of our cohort. The use of gradient echo cine sequences, which are less susceptible to metal artefacts, can reduce rates of non-diagnostic imaging.

## Funding

The study was funded by the NIHR Cardiovascular BRU. CER is supported by the British Heart Foundation.

